# Structural Alteration of Medial Temporal Lobe Subfield in the Amnestic Mild Cognitive Impairment Stage of Alzheimer's Disease

**DOI:** 10.1155/2022/8461235

**Published:** 2022-01-24

**Authors:** Pan He, Hang Qu, Ming Cai, Weijie Liu, Xinyi Gu, Qiang Ma

**Affiliations:** ^1^Department of Neurology, Affiliated Zhongshan Hospital of Dalian University, Dalian, 116001 Liaoning Province, China; ^2^Department of Radiology, Medical Imaging Center, The Affiliated Hospital of Yangzhou University, Yangzhou University, Yangzhou Jiangsu, China

## Abstract

**Objective:**

Volume reduction and structural abnormality is the most replicated finding in neuroimaging studies of Alzheimer's disease (AD). Amnestic mild cognitive impairment (aMCI) is the early stage of AD development. Thus, it is necessary to investigate the link between atrophy of regions of interest (ROIs) in medial temporal lobe, the variation trend of ROI densities and volumes among patients with cognitive impairment, and the distribution characteristics of ROIs in the aMCI group, Alzheimer's disease (AD) group, and normal control (NC) group.

**Methods:**

30 patients with aMCI, 16 patients with AD, and 30 NC are recruited; magnetic resonance imaging (MRI) brain scans are conducted. Voxel-based morphometry was employed to conduct the quantitative measurement of gray matter densities of the hippocampus, amygdala, entorhinal cortex, and mammillary body (MB). FreeSurfer was utilized to automatically segment the hippocampus into 21 subregions and the amygdala into 9 subregions. Then, their subregion volumes and total volume were calculated. Finally, the ANOVA and multiple comparisons were performed on the above-mentioned data from these three groups.

**Results:**

AD had lower GM densities than MCI, and MCI had lower GM densities than NC, but not all of the differences were statistically significant. In the comparisons of AD-aMCI-NC, AD-aMCI, and AD-NC, the hippocampus, amygdala, and entorhinal cortex showed differences in the gray matter densities (*p* < 0.05); the differences of mammillary body densities were not significant in the random comparison between these three groups (*p* > 0.05). The hippocampus densities and volumes of the subjects from the aMCI group and the AD group were bilaterally symmetric. The gray matter densities of the right side of the entorhinal cortex inside each group and the hippocampus from the NC group were higher than those of the left side (*p* < 0.05), and the gray matter densities of the amygdala and mammillary body were bilaterally symmetric in the three groups (*p* > 0.05). There were no gender differences of four ROIs in the AD, aMCI, and NC groups (*p* > 0.05). The volume differences of the hippocampus presubiculum-body and parasubiculum manifest no statistical significance (*p* > 0.05) in the random comparison between these three groups. Volume differences of the left amygdala basal nucleus, the left lateral nucleus, the left cortical amygdala transitional area, the left paravamnion nucleus, and bilateral hippocampal amygdala transition area (HATA) had statistical differences only between the AD group and the NC group (*p* < 0.05).

**Conclusion:**

Structural defects of medial temporal lobe subfields were revealed in the aMCI and AD groups. Decreased gray matter densities of the hippocampus, entorhinal cortex, and amygdala could distinguish patients with early stage of AD between aMCI and NC. Volume decline of the hippocampus and amygdala subfields could only distinguish AD between NC.

## 1. Introduction

Alzheimer's disease (AD), with a long incubation period, is an irreversible neurological degenerative disease. AD was divided into three stages: preclinical stage, mild cognitive impairment (MCI) stage, and dementia stage. MCI is the early stage of AD development. During this stage, patients suffer from slight memory decline and cognitive impairment, but their ability to deal with basic tasks in life has not been affected, and these symptoms do not reach the diagnostic criteria of AD. MCI chiefly contains two types: amnestic mild cognitive impairment (aMCI) and nonamnestic mild cognitive impairment. To be specific, aMCI patients, who suffer primarily from episodic memory decline, are the high-risk group that will most commonly progress into AD patients. It is widely acknowledged that treatment effects shown on patients during AD stage are not satisfactory. However, receiving treatment during the MCI stage will effectively postpone the development of AD [1, 2]. Therefore, during MCI stage, the atrophy of the brain structure and the choice of biomarkers are of immense clinical significance to make early prediction of AD, accurate diagnosis of AD, and judgement in the development trend of AD. In this study, gray matter of the medial temporal lobe and its surrounding areas were selected as the region of interest. These gray matters were all fundamental nuclei in the Papez loop and extremely correlated with episodic memory, including the hippocampus, amygdala, entorhinal cortex, and mammillary body, mostly located in the medial temporal lobe. Wirt and Hyman [3] supposed that the hippocampus, entorhinal cortex, and amygdala are the basis of encoding and storing memory. The mammillary body is part of the diencephalon; as part of the Papez circuit, it would result in episodic memory impairment if damaged [4], which also has certain research significance. Due to the previous studies on the changes in the gray matter of the medial temporal lobe in aMCI patients, different research methods were adopted, including different versions of MCI diagnostic guidelines and different brain structure definition criteria, as well as the inherent complexity of gray matter in the medial temporal lobe and the limitations of imaging methods. The conclusion of MTL atrophy in aMCI patients has been highly controversial. In this study, the hippocampus, entorhinal cortex, amygdala, and MB were selected as regions of interest (ROIs), and voxel-based morphometry was utilized to conduct the quantitative measurement of the densities of the hippocampus, amygdala, entorhinal cortex, and MB gray matter. Meanwhile, FreeSurfer was used to automatically segment the hippocampus and amygdala and then calculate their subregion volume and total volume. Through this experimental design, we aimed to investigate the structural changes in gray matter in MTL related to memory function, the symmetry and gender differences of ROIs in each group, and the correlation of structural alteration with cognitive dysfunction in aMCI and AD.

## 2. Material and Methods

### 2.1. Subjects Included

Patients from the Memory Clinic of Affiliated Zhongshan Hospital of Dalian University (Dalian, China) from February 2018 to August 2018 were selected. The AD group consisted of 16 subjects, 8 males and 8 females at the age range from 60 to 80, with the average age of 70. They were all right-handed, and their courses of disease were ranged from 2 years to 4 years, with an average of 3 years; the MMSE scores were ranged from 12 to 20 points; and the MoCA scores were ranged from 10 to 17 points, with the clinical dementia rating (CDR) score of 1 point. The aMCI group involved 30 right-handed subjects, 15 males and 15 females at the age range from 60 to 80, with the average age of 65; the courses of disease were ranged from 3 months to 18 months, with an average of 8 months; the MMSE scores were ranged from 24 to 28 points; the MoCA scores were ranged from 18 to 26 points; and the CDR scores were 0.5 points. The clinical symptoms featured the degeneration of episodic memory and recent memory. Then, another group of 30 subjects matching the age, sex, and educational status of those from the aMCI group and the AD group was selected as the NC group: 15 males and 15 females at the age range from 60 to 80, with the average age of 67. In this study, subjects in the AD group were collected according to the diagnostic criteria of AD combined with clinical symptoms and cognitive function scales of patients. Most of them refused to accept the examination, such as amyloid PET or lumbar puncture. Therefore, there is a lack of comprehensive data such as amyloid-PET.

### 2.2. Inclusion Criteria

Preliminary selection was done via the following neuropsychological scales [5]: Mini-Mental State Examination (MMSE), Montreal Cognitive Assessment (MoCA), Activities of Daily Living Evaluation (ADL), Hachinski Ischemic Scale, Frontal Assessment Battery (FAB), Hamilton Anxiety Scale (HAMA), and Hamilton Depression Scale (HAMD).

The aMCI group inclusion criteria referred to the diagnostic guidelines for mild cognitive impairment due to Alzheimer's disease developed by the National Aging Institute (NIA) and the Alzheimer's Society (AA) in 2011 [6], Petersen et al. in 1999 [7], and the diagnostic criteria proposed by the aMCI Working Group of the European AD Association and the American Diagnostic and Statistical Manual of Mental Disorders in 2006 [8]. The patient or his family members objectively reflected memory loss that was not equivalent to his age, the course of disease was >3 months, and the onset was insidious and progress was slow, with notable episodic memory delayed memory impairment in early stage. MMSE score: ≥24 points for years of education greater than or equal to 7 years, ≥20 points for years of education less than 7 years, and ≥17 points for illiteracy; MoCA score: 19-25 points; CDR = 0.5 points; the ability of daily living was not impaired ADL < 18.

The AD group inclusion criteria could be learnt from the *Diagnostic Guidelines* [9] published in April 2011 by the Alzheimer's Association and the wholly optimized version of the above-mentioned *Diagnostic Guidelines* [10] done by the International Working Group in Lancet Neurol in 2014.The patient or his family members objectively reflected progressive deterioration of memory function or other cognitive functions over 6 months and progressively aggravated. MMSE score: <20 points, MOCA score: 11-20 points, Hachinski < 4 points, Fab ≥ 12; CDR = 1 point; impaired activities of daily living ADL > 26 points.

In addition, the NC group of healthy subjects, who matched the age, sex, and educational status of those from the aMCI group and the AD group, with normal memory and no severe diseases in the nervous system or other systems, was included.

### 2.3. MRI Examination Method

3.0 T Superconducting Magnetic Resonance Scanner (Siemens, TIM Trio, Erlangen, Germany) was employed to conduct MRI scanning: with a 12-channel standard head coil enhancing the signal-to-noise ratio, whole brain scan (from the calvarium to the foramen magnum region) with the 3D-MP-RAGE sequence was performed on all subjects to obtain whole-brain 3D-T1WI structure images. The scan parameters were as follows: repetition time (TR) = 2530 ms, echo time (TE) = 2.22 ms, flip angle (FA) = 7°, MATRIX = 224 × 224, field of view (FOV) = 224 mm × 224 mm, VS = 1 mm × 1 mm × 1 mm, and scanning time = 5 min and 28 sec; slice thickness = 0.9 mm, slice distance = 0 mm, and slice numbers = 176.

### 2.4. MRI Data Acquisition and Processing

The scalp part and the neck part of 3D-T1WI were removed. The VBM process was as follows: Statistical Parametric Mapping 12 (http://www.fil.ion.ucl.ac.uk/spm/software/spm12/) was employed to analyze the MRI data [11, 12]; through the DARTEL algorithm, each participant's images were normalized into MNI152 space provided by the Montreal Neurological Institute, and in the meantime, the normalized images were modulated in order to make sure that relative gray and white matter volumes were well preserved following spatial normalization. Eventually, these images were smoothed with an 8 mm full-width-at-half-maximum Gaussian kernel. The hippocampus, entorhinal cortex, amygdala, and MB were selected as ROIs and were used to measure the densities of the ROIs.

Via FreeSurfer, the subregions of the hippocampus and amygdala were automatically segmented, and then, the volumes of their subregions were calculated. Additionally, through the FreeSurfer 6.0 image analysis suite, cortical reconstruction and segmentation were conducted. With the previously defined in vivo and ex vivo hippocampus and amygdala atlases to determine subregion characteristics, contrast differences between subregions were interrogated. Selecting the 3 T MRI flag and multispectral segmentation in FreeSurfer could optimize the procedure. 21 hippocampus subregions and 9 amygdala subregions were computed.

**Figure 1:**
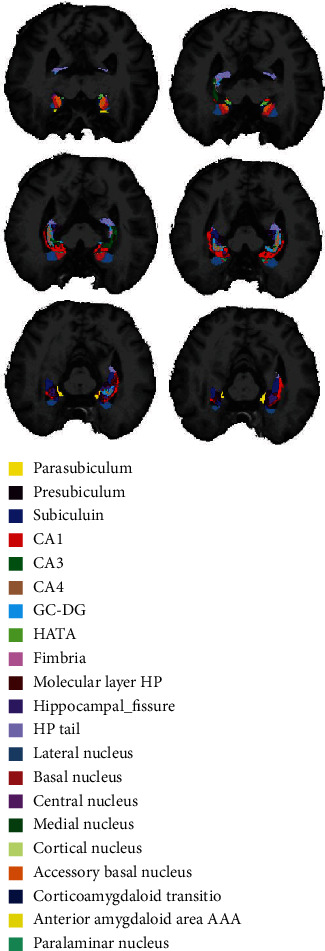
Segmentation template of the hippocampus and amygdala.

The hippocampus consisted of 21 subregions: subiculum-head, subiculum-body, presubiculum-head, presubiculum-body, parasubiculum, hippocampal-fissure, CA1 head, CA1 body, CA3 head, CA3 body, CA4 head, CA4 body, molecular layer-head, molecular layer-body, granular cell layer-molecular layer-dentated gyrus head (GC-ML-DG-head), granular cell layer-molecular layer-dentated gyrus body (GC-ML-DG-body), fimbria, hippocampal amygdala transition area (HATA), hippocampal-tail, whole hippocampal-head, whole hippocampal-body, and whole hippocampus. The amygdala included 9 subregions: lateral, basal, accessory basal, anterior amygdala area, central, medial, cortical, corticoamygdaloid transition area, and paralaminar nucleus. Visualization of the hippocampal and amygdala subregions is shown in Figure 1.

### 2.5. Statistical Analysis

SPM12 of the MATLAB platform was applied to conduct ANOVA test and multiple comparisons on bilateral ROIs' gray matter (GM) densities of the subjects from the three groups. Via the use of the general linear model (GLM) in SPM12, the assessment of morphological differences between side differences and sex differences in bilateral ROIs' GM densities was done. The paired-sample *t*-test was utilized to detect the side difference in the four ROIs' GM densities between and inside each group, and two independent-sample *t*-tests were used to detect the sex difference in the four ROIs' GM densities between and inside each group. In order to exclude global nuisance effects, age and total intracranial volumes (TIV) of each participant were entered into the design matrix as covariance.

All extracted subfield volumes were systematically inspected visually and measures exported to SPSS24. ANOVA was used to investigate group-wise differences in substructure-composite volumes and across all groups. Additional post hoc of *t*-test was used to compare between-group differences (aMCI vs. AD, aMCI vs. NC, and AD vs. NC) for each substructure-composite and hemisphere independently. Age, sex, and TIV were entered as covariates throughout. Multiple Comparison Correction was performed using False Discovery Rate (FDR) correction.

## 3. Results

- After chi-square test and ANOVA test were performed, respectively, on the sex and age of the subjects from the three groups, it was discovered that the age difference and the sex difference in the simultaneous comparison between the three groups were of no statistical significance (*p* > 0.05). Apart from this, through the ANOVA test, it could be seen that the difference in MMSE and MoCA in the simultaneous comparison between the three groups was of statistical significance (*p* < 0.05), and the scores gradually increased in each group (Table 1).
- Comparison between side differences and sex differences in bilateral ROIs' GM densities in each group are shown in Table 2.

Side differences: the gray densities of the right side of the entorhinal cortex inside each group and hippocampus from the NC group were higher than those of the left side, and this difference was statistically significant (*p* < 0.05), and densities of the amygdala and mammillary body were bilaterally symmetric (*p* > 0.05). Sex differences: four ROIs in each group did not show any sex differences (*p* > 0.05).

(iii) The comparison of ROIs' GM densities between the three groups are shown in Table 3. The differences of the densities of the bilateral hippocampus, amygdala, and entorhinal cortex in the comparisons of AD-aMCI-NC, AD-aMCI, and AD-NC were statistically significant (*p* < 0.05) but not that obvious in the comparison of aMCI-NC (*p* > 0.05); the differences of the density of the mammillary body were not notable in the random comparison between these three groups (*p* > 0.05).
(iv) The comparison of the subregion volumes of the hippocampus and amygdala between the three groups are shown in Tables 4, 5, 6, and 7. From Tables 4, 5, 6, and 7, it can be seen that the bilateral volume differences of the hippocampus presubiculum-body and the hippocampus parasubiculum in the random comparison between the AD, aMCI, and NC groups were not statistically significant (*p* > 0.05), and the volume differences in the left side of the amygdala basal, accessory basal, corticoamygdaloid transition area (CAT), paravamnion nucleus, and bilateral hippocampus amygdaloid transition area (HATA) were only statistically significant in distinguishing the AD group and the NC group (*p* < 0.05).

## 4. Discussion

Mild cognitive impairment is the early stage of Alzheimer's disease. MCI patients suffer from slight memory and intellectual impairment, and the degree of the damage to their cognitive function exceeds the cognitive state of the ones with the same age and education background. However, their general cognitive function and the ability to deal with basic tasks in life have not declined obviously, and symptoms do not meet the diagnostic criteria of AD. MCI mainly includes amnestic mild cognitive impairment (aMCI) and nonamnestic mild cognitive impairment. Notably, aMCI patients chiefly featured by episodic memory damage belong to the high-risk group that can be easily progressed into AD patients. Currently, the research focused on the diagnosis of MCI indicates that the biomarkers in the cerebrospinal fluid (tau protein and A*β* protein) are the most valuable predictors that can evaluate the time when elderly people or MCI patients progress into AD patients. Nevertheless, in clinical conditions, it is not easy to obtain CSF since obtaining CSF can cause invasion to patients; additionally, the costs of molecular imaging are expensive. The nervous system abnormality of aMCI was first specifically involved in the MTL initially, leading to the earliest and most obvious damage to the episodic memory of the patients. Through studying 145 MCI patients, Whitwell et al. [13] found out that different subtypes of MCI led to atrophy in different regions of the brain. Additionally, not only the single-domain aMCI group but also the multidomain aMCI group suffered from gray matter loss primarily in MTL. In the meantime, it could be observed that in the multidomain group, gray matter loss extended into the posterior lateral, basal temporal lobe. Moreover, past research [14] substantiated that MTL atrophy is the most reliable imaging evidence that can predict the progression from aMCI to AD. Consequently, observing and analyzing the degree of MTL atrophy with structural MRI could diagnose MCI objectively and accurately and predict the progression from MCI to AD.

Previous researches investigated the degree of MTL atrophy through manual delimitation; however, manual delimitation was quite sensitive to the variability of individual subject and was heavily affected by the subjectivity of researchers. In recent years, the development of high-resolution MRI technology and automatic segmentation technology makes it possible to investigate the correlation between the degree of MTL atrophy and patients with cognitive impairment, analyze the symmetry and sex difference of ROIs in each group, and probe into the variation trends and clinical applications among patients with cognitive impairment. Gill et al. [15] used T1-weighted and diffusion-tensor magnetic resonance imaging (DTI) to investigate if regional micro- and macrostructural brain properties were associated with impulse dyscontrol symptoms in older adults with normal cognition, mild cognitive impairment, and Alzheimer's disease (AD). The results suggest that impulse dyscontrol symptoms act as an early manifestation of AD and significantly lower cortical thickness in the parahippocampal gyrus. Through comparing four analytical technologies of structural MRI in AD patients including manual delimitation, automatic volume measurement, cortex thickness analysis, and VBM, Clerx et al. [16] aimed to investigate the optimized technology to diagnose early AD. What was proposed was that manual delimitation and VBM were both the most preferred methods to assess atrophy degree of gray matter of AD patients and to manifest MTL atrophy of early AD most remarkably. Meanwhile, the research indicated that FreeSurfer could reflect the atrophy of brain tissue during early AD but could only display that statistical significance existed in the posterior parietal cortex (PPC), and the difference between MTL of AD patients and that of normal controls was not obvious. In addition, measurements of the cortex thickness were less sensitive in the MCI stage—they could merely discover atrophy in MTL and PPC in the later stage of AD. Mulder et al. [17] proposed that the reproducibility of FreeSurfer was superior to that of manual delimitation and FIRST. Marizzoni et al. [18] investigated the reproducibility of automatically segmented hippocampal subfields and supposed that the test-retest reproducibility of hippocampal subfield volume segmentations is significantly improved if two within-session T1 anatomical scans are averaged relative to using a single T1 acquisition.

Although FreeSurfer could detect the degree of the decrease in the volume of MTL of patients with early AD, MTL atrophy was not the specific change of AD. Since temporal lobe epilepsy, depression, and schizophrenia could also cause MTL atrophy, making the diagnosis of aMCI be based only on the volume change of MTL which could lead to false positive rate. In addition, senile plaques and neurofibrillary tangles (NFT) could give rise to neuronal loss and axonal demyelination that lead to the decline in the volume of MTL at the stage of aMCI; however, at the same time, the decrease in the volume of MTL at the stage of aMCI was covered to some extent due to compensatory hyperplasia of astrocyte, so making the diagnosis of aMCI be only according to the outcomes of volume measurement could also lead to false negative diagnosis. Therefore, in this study, FreeSurfer was used and VBM was combined to measure the gray matter density of ROIs, which greatly increased the reliability of research results.

Even though MTL atrophy among MCI patients has been widely reported internationally, relative research results were controversial because of different research methods, delimitation criteria, and diagnostic guidelines of MCI. In this study, the latest international diagnostic guidelines (IWG-2 and 2017AAN) were adopted, and the aMCI group, AD group, and NC group were collected. The VBM-DARTEL algorithm was used to calculate the GM densities of the hippocampus, amygdala, entorhinal cortex, and MB of each group, and the distribution characteristic of the GM densities of these four ROIs was analyzed. The result showed that the difference in the GM densities of the hippocampus, amygdala, and entorhinal cortex between the AD group and the aMCI group and between the AD group and the NC group was of statistical significance (*p* < 0.05), while the difference in the GM densities of the four ROIs between the aMCI group and the NC group was of no statistical significance (*p* > 0.05). This indicated that the decrease in the GM densities of the four ROIs could not be set as the ideal indicator to diagnose aMCI, meaning that it was quite hard to accurately distinguish aMCI patients from healthy elderly people at the individual level. Nonetheless, in distinguishing between aMCI patients and patients with early AD, the decrease in the GM densities of the hippocampus, amygdala, and entorhinal cortex was rather helpful, which corresponded to the viewpoint held by Kunst et al. [19] that the difference in the GM densities of the ROIs between the MCI group and the NC group was not statistically significant in the research into atrophy degree of GM in MTL of early AD and MCI through VBM and SBM. But some research argued that [20, 21] the hippocampus, amygdala, and entorhinal cortex could be used in distinguishing between MCI patients and the subjects from the NC group, which did not correspond to the outcome in this study. This paper thought that the differences of 4 ROIs between subjects from the MCI group and the ones from the NC group were not obvious in this study due to the following reasons. Firstly, MCI was of clinical heterogeneity, and the possibility of progression from aMCI into AD was the maximum, with the annual progression rate of 10%-15%. Because some researchers did not classify MCI into many subtypes and the diagnostic criteria of MCI, what they adopted was different, and the accuracy of their outcomes was affected. Secondly, research has also found that MTL only underwent metabolic changes at the stage of aMCI [22], which had not led to obvious atrophy in the brain structure or atrophy, probably since the astrocyte experienced compensatory hyperplasia-metabolic changes which preceded structural changes, leading to no structural imaging changes of MCI patients. These hypotheses need to be confirmed by further large sample studies.

In past studies, few researches on pathological changes of MB were reported internationally. Through manual delimitation, Pian et al. [23] thought that the MB atrophy in the simultaneous comparison between three groups was statistically different. In this study, the difference of the GM densities of MB measured by VBM was of no statistical significance between any two of the three groups, so MB atrophy could not be used in distinguishing the subjects between all the three groups, which matched the research outcomes obtained by Zhu [24]. Because MB with small volume and complicated inner structure was varied individually and MB atrophy could occur in such diseases as liver failure, heart failure, sleep apnea syndrome (SAS), congenital central hypoventilation syndrome (CCHS), and Wernicke-Korsakoff brain disease, there was still a lack of specificity to diagnose MCI or AD based on the atrophy degree of MB. In addition, located at the border of the blood supply area of the posterior cerebral artery and the posterior communicating artery, MB had a low capacity of blood supply; the atherosclerotic plaque in the internal carotid artery and posterior cerebral artery tended to fall off to cause infarction inside the MB arteriole—these two reasons led MB to undergo ischemia easily. Moreover, it could also be assumed on the basis of these two reasons that the degree of correlation between MB and vascular dementia could be higher.

Through relative research, Long et al. [25] verified that aging and neurodegenerative diseases could possibly affect the dominant hemisphere first, and hemispheric asymmetry was probably one of the indicators of high-risk morphological variations to predict the progression of MIC patients into AD patients. Nevertheless, the research outcome of side differences and sex differences of GM structures relative to memory function of bilateral MTL in the aMCI group, AD group, and NC group was still controversial. Derflinger et al. [26] supposed that brain atrophy of AD patients was of asymmetry but not of lateralization. Through VBM, Minkova et al. [27] and Son et al. [28] put forward that among aMCI patients, the atrophy degree of the right hippocampus was more obvious than that of the left one; however, also through VBM, Ota et al. [29] and Ferreira et al. [30] believed that among aMCI patients, GM density of the left hippocampus underwent a much more obvious decline than that of the right one. Zhou et al. [31] deemed that among dementia patients, the right entorhinal cortex atrophied more obviously than the left one, which could be used in distinguishing between MCI patients and healthy elderly people. Nevertheless, since the structure of the entorhinal cortex varied vastly among individuals and manual delimitation was affected by the pulse of the Willis loop artery and image artifacts of CSF in the suprasellar cistern, errors tended to occur. Zanchi et al. [32] and Goerlich [33] held opposite opinions over the size of bilateral amygdala among MCI patients: the former believed that the right amygdala atrophied more notably than the left one, and the latter thought that the left amygdala atrophied more obviously than the right one. In this study, VBM and FreeSurfer were used to, respectively, measure the densities and volumes of the four ROIs. The outcome showed that the hippocampus densities and volumes of the subjects from the aMCI group and AD group were bilaterally symmetric. However, among the subjects of the NC group, the right hippocampus was larger than the left one, and among the subjects across the three groups, the right entorhinal cortex was larger than the left one—the difference was of statistical significance (*p* < 0.05). This corresponded to the viewpoint about the variation trend of cerebral hemisphere asymmetry among MCI patients proposed by Zhou et al. [31] in their research. Additionally, the amygdala and MB were bilaterally symmetric among the subjects in the three groups. Rezzani et al. [34] supposed that frontal and medial paralimbic brain regions are larger in women, whereas the hypothalamus, amygdala, and angular gyrus seem to be larger in men. Grabowska [35] supposed that in females, the volume of the corpus callosum and temporal and parietal regions (surrounding the Sylvian fissure) engaged in language processing is comparatively larger, whereas males have a larger parietal cortical area associated with visual-spatial function. But the results of this study suggest that the four ROIs were of no sex differences (*p* > 0.05).

The hippocampus, a pair of complex and heterogeneous structures composed of the subregions that are interacted and interconnected with each other, is connected to other brain structures through the entorhinal cortex. In the early stage of cognitive impairment, whether the hippocampus is affected by pathological changes is still debated. Due to its deep location inside the brain and its complex delimitation, in the past neuroimaging study, during the process of hippocampus modeling, the hippocampus was usually considered as a pair of homogeneous structures without any interrelations, and useful information hidden in its subregions was ignored. Nevertheless, according to the recent studies of rodents and primates, hippocampus subregions could offer great amounts of information. Different hippocampus subregions possessed different memory functions and segmenting subregions through high-resolution MRI of the hippocampus could improve the sensitivity of detecting lesions in hippocampus subregions. Most studies divided the hippocampus into three subregions: head, body, and tail. And some researches divided the hippocampus into another three regions based on functions: emotion subregion, cognition subregion, and perception subregion. With the combination of the ANDI atlas and Bayesian atlas, Iglesias et al. [36] employed MRI with ultrahigh-resolution MRI (0.13 mm) to construct the statistical atlas of the hippocampus substructure level and segment the hippocampus into 13 subregions. In this study, on the basis of the 13 subregions segmented in the study of Iglesias et al., FreeSurfer was adopted to segment the hippocampus into 21 subregions, which effectively reflect the subtle changes of the hippocampus of aMCI patients. The result indicated that the volume difference of all the subregions except the presubiculum-body and parasubiculum in the bilateral hippocampus between the AD and MCI groups and between the AD and NC groups was of statistical significance (*p* < 0.05). However, no statistical significance existed between the MCI group and the NC group when it came to the volume difference of the 21 subregions in the bilateral hippocampus (*p* > 0.05); all the hippocampus subregions could not be used in distinguishing between aMCI patients and healthy elderly people.

Neuroimaging study has vindicated that the amygdala is a complex composed of subnucleus with heterogeneous structures and functions, not a purely homogeneous structure. After Amunts et al. [37] employed cytoarchitectonics to divide the amygdala into the lateral basal (LB), centromedial (CM), and superficial (SF), the amygdala was constantly divided in this way in the past studies. LB receives the input from the auditory system; in particular, the thalamus and brain cortex both take part in the transmission of conditioned stimuli. Neural coding inside LB was correlated with fear memory caused by these sensory stimuli. These stimuli can emit signals of threat values of the stimuli and regulate memory coding and sensory processing in other brain regions. CM probably plays a crucial role in generating behavioral responses, and through the projection to the brainstem, hypothalamus, and corpus striatum, it could fulfill this function. SF, which has a broad bidirectional connection with the hippocampus, entorhinal cortex, insular lobe, and septal nulcei, is involved in the detection of remarkable emotional stimuli and dealing with information related to society. Based on the ANDI atlas and the ABIDE atlas, Saygin et al. [38] employed ultrahigh-resolution MRI to construct an atlas in which the amygdala was divided into 9 subregions. Then, this atlas was published in the software Distribute of FreeSurfer. It was worth noting that this very atlas was able to analyze sMRI data with any contrast. In this study, with the assistance of FreeSurfer, on the basis of the three subregions segmented in the previous research, the amygdala was divided into nine subregions, and then, their volumes were calculated, which more precisely and objectively analyzed the variation of amygdala volumes of aMCI patients. As the result indicated, the volume differences of the left amygdala basal nucleus, the left lateral nucleus, the left cortical amygdala transitional area, the left paravamnion nucleus, and the bilateral hippocampal amygdala transition area (HATA) possessed statistical differences only in distinguishing the AD group and the NC group (*p* < 0.05), and the difference in the volumes of other subregions between the AD and aMCI groups and between the AD and NC groups was of statistical significance (*p* < 0.05). However, the difference in the volumes of the nine subregions between the aMCI group and the NC group was of no statistical significance (*p* > 0.05); all the amygdala subregions could not be used to distinguish between the subjects in the aMCI group and the NC group.

## 5. Conclusion

In this study, through the latest international diagnostic guidelines, the aMCI, AD, and NC groups were collected. Combining VBM and automated subregion analysis, we found that decrease in the GM densities of the hippocampus, amygdala, and entorhinal cortex was rather helpful in distinguishing between aMCI and patients with early AD; however, it was quite hard to accurately distinguish aMCI patients from healthy elderly people. Different from normal control, the hippocampus densities and volumes of the aMCI group and the AD group were bilaterally symmetric, corresponding to the viewpoint of the variation trend of cerebral hemisphere asymmetry among MCI patients. Most subfields of the hippocampus and amygdala could distinguish between the AD and aMCI groups and between the AD and NC groups; however, there is no statistical difference in subfields of the hippocampus and amygdala between aMCI and NC. Through the analysis which investigated the variation trend and application value of structural alteration of aMCI and AD patients, we tried to find reliable imaging evidence that can predict the early onset of AD. Our results have benefits to diagnose aMCI objectively and accurately and predict the progression from aMCI to AD; however, we failed to achieve the structural imaging indicators with high sensitivity and specificity that could successfully distinguish the aMCI and AD.

Admittedly, this study still had its own limitations. First, the sample size was extremely small and could not represent the characteristics of MTL atrophy of the overall population of aMCI patients. Second, molecular imaging or functional MRI could detect metabolic changes in brain tissue before its structural changes took place among aMCI patients. Furthermore, amyloid-PET is the golden standard to diagnosticate AD in nuclear medicine. If combined with DTI, functional MRI, FDG-PET, PiB-PEt, multimodal MRI, or hyperbrain network, more valuable predictors would be discovered. And test-retest is necessary in future studies to increase the accuracy and reproducibility of results. Therefore, in the future researches about brain tissue changes of aMCI patients, enlarging the sample size and broadening the research perspective will bring about more research significance in the early diagnosis of aMCI.

## Figures and Tables

**Table 1:** Comparison of the basic information of the experimental subjects.

	AD	aMCI	NC	Standard value	*p* value
Number	16	30	30		
Sex (M/F)	8/8	15/15	15/15	1.88	0.42
Average age	70.68 ± 4.56	66.86 ± 5.75	64.63 ± 5.67	1.76	0.19
MMSE	16.56 ± 4.25	27.25 ± 1.23	28.69 ± 1.03	159.37	<0.05^∗^
MoCA	10.97 ± 3.02	20.56 ± 2.65	25.48 ± 2.01	174.68	<0.05^∗^

**Table 2:** Statistical analytical results of side differences and sex differences in the subjects' GM densities of bilateral ROIs inside each group. Side differences: the densities of the right side of the entorhinal cortex inside each group and the hippocampus from the NC group were higher than those of the left side, this difference was statistically significant (*p* < 0.05), and densities of the amygdala and mammillary body were bilaterally symmetric. Sex differences: four ROIs in each group did not show any sex differences (*p* > 0.05).

	AD		MCI		NC	
	Side	Sex	Side	Sex	Side	Sex
Hippo-T	-0.495	0.215	-0.237	0.076	-3.473	0.598
Hippo-P	0.632	0.835	0.814	0.369	0.002^∗^	0.555
Amygdala-T	0.152	0.016	0.421	-0.816	-0.009	-0.28
Amygdala-P	0.883	0.987	0.677	0.422	0.993	0.781
EC-T	-4.824	0.45	-4.763	-0.836	-6.985	0.471
EC-P	0.001^∗^	0.665	0.1 ×10^−5^^∗^	0.41	0.1 ×10^−7^^∗^	0.641
MB-T	-1.389	0.45	-0.814	1.417	-1.846	0.708
MB-P	0.198	0.164	0.422	0.168	0.076	0.485

**Table 3:** Results of the ANOVA test and multiple comparisons of bilateral ROIs' GM densities of the three groups. The differences of the densities of the bilateral hippocampus, amygdala, and entorhinal cortex in the comparisons of AD-MCI-NC, AD-MCI, and AD-NC were statistically significant (*p* < 0.05) but not that obvious in comparison with MCI-NC (*p* > 0.05); the differences of the density of the mammillary body were not notable in the random comparison between these three groups (*p* > 0.05).

	AD-MCI-NC		AD-MCI		AD-NC		NC-MCI	
	*f* value	*p* value	*t* value	*p* value	*t* value	*p* value	*t* value	*p* value
L-AMYG	6.6793	0.0023^∗^	-3.1442	0.0033^∗^	-3.569	0.001^∗^	-0.1664	0.8684
R-AMYG	8.423	0.0006^∗^	-3.797	0.0005^∗^	-3.5467	0.0011^∗^	0.1902	0.8499
L-EC	4.8039	0.0114^∗^	-2.7972	0.0081^∗^	-2.8397	0.0074^∗^	-0.0532	0.9577
R-EC	4.8778	0.0107^∗^	-2.9915	0.0049^∗^	-2.6157	0.0129^∗^	-0.0082	0.9935
L-Hippo	7.3448	0.0013^∗^	-3.4393	0.0015^∗^	-3.5846	0.001^∗^	-0.3777	0.7071
R-Hippo	13.4588	1.3×10^−5^^∗^	-4.7372	3.1×10^−5^^∗^	-4.3955	0.1 ×10^−3^^∗^	0.499	0.6197
L-MB	0.1364	0.8727	-0.1186	0.9062	0.2275	0.8213	-0.5567	0.58
R-MB	0.3072	0.7366	0.7232	0.4741	0.2941	0.7704	0.5729	0.569

**Table 4:** ANOVA test and multiple comparison results of the subregion volumes of the left hippocampus between the three groups.

Left	AD-MCI-NC		AD vs. MCI		AD vs. NC		NC vs. MCI	
	*f* value	*p* value	*t* value	*p* value	*t* value	*p* value	*t* value	*p* value
SUB-head	4.874	0.0109^∗^	-2.636	0.0122^∗^	-3.681	0.8 × 10^−3∗^	-0.467	0.6424
SUB-body	7.178	0.0016^∗^	-3.314	0.0021^∗^	-3.858	0.5 × 10^−3∗^	-0.606	0.5473
CA1-head	3.833	0.027^∗^	-2.254	0.0302^∗^	-4.717	0.4 × 10^−4∗^	-0.696	0.4897
CA1-body	5.873	0.0046^∗^	-3.060	0.0041^∗^	-3.796	0.6 × 10^−3∗^	-0.175	0.8615
CA3-head	3.272	0.0447^∗^	-2.136	0.0393^∗^	-3.218	0.0029^∗^	-0.810	0.4217
CA3-body	4.815	0.0114^∗^	-2.744	0.0093^∗^	-3.15	0.0035^∗^	-0.862	0.3926
CA4-head	4.664	0.013^∗^	-2.522	0.0161^∗^	-3.749	0.7 × 10^−3∗^	-1.202	0.2347
CA4-body	6.328	0.0032^∗^	-2.935	0.0057^∗^	-4.581	0.1 × 10^−3∗^	-0.526	0.6011
HIPPO-fissure	5.622	0.0057^∗^	-3.308	0.0021^∗^	-2.499	0.0176^∗^	-1.044	0.3013
PRE-head	5.186	0.0083^∗^	-2.717	0.01^∗^	-3.704	0.0008^∗^	-0.493	0.6241
PRE-body	1.12	0.3328	-1.442	0.1578	-1.266	0.2146	-0.293	0.7705
PARA	0.27	0.7642	-0.111	0.912	-0.649	0.5211	0.615	0.5415
ML-head	4.684	0.0128^∗^	-2.497	0.0171^∗^	-4.633	0.1 × 10^−3∗^	-0.762	0.4496
ML-body	6.005	0.0042^∗^	-2.899	0.0063^∗^	-4.114	0.2 × 10^−3∗^	-0.302	0.7638
GC-ML-DG-head	4.408	0.0163^∗^	-2.461	0.0186^∗^	-3.951	0.4 × 10^−3∗^	-1.011	0.3167
GC-ML-DG-body	5.327	0.0074^∗^	-2.672	0.0112^∗^	-4.296	0.1 × 10^−3∗^	-0.42	0.6762
FIM	2.432	0.0964	-1.837	0.0742	-2.649	0.0123^∗^	-0.041	0.9676
HATA	1.501	0.2311	-1.214	0.2324	-3.335	0.0021^∗^	0.318	0.7521
Whole_HIPPO_head	4.179	0.0199^∗^	-2.356	0.0239^∗^	-4.511	0.1 × 10^−3∗^	-0.682	0.498
Whole_HIPPO_body	5.768	0.0051^∗^	-2.814	0.0078^∗^	-4.185	0.2 × 10^−3∗^	-0.442	0.6603
TAIL	7.476	0.0012^∗^	-3.318	0.002^∗^	-4.406	0.1 × 10^−3∗^	-0.337	0.7376
Whole_hippocampus	5.455	0.0066^∗^	-2.702	0.0103^∗^	-4.707	0.4 × 10^−4∗^	-0.588	0.5593

**Table 5:** ANOVA test and multiple comparison results of the subregion volumes of the right hippocampus between the three groups.

Right	AD-MCI-NC		AD vs. MCI		AD vs. NC		NC vs. MCI	
	*f* value	*p* value	*t* value	*p* value	*t* value	*p* value	*t* value	*p* value
SUB-head	14.118	9.1 × 10^−6∗^	-4.86	2.2 × 10^−5∗^	-4.92	2.3 × 10^−5∗^	0.947	0.3482
SUB-body	15.224	4.3 × 10^−6∗^	-4.982	1.5 × 10^−5∗^	-5.11	1.3 × 10^−5∗^	0.056	0.9556
CA1-head	15.507	3.6 × 10^−6∗^	-4.892	1.97 × 10^−5∗^	-5.211	9.9 × 10^−6∗^	0.989	0.3271
CA1-body	10.02	0.0002^∗^	-5.467	3.3 × 10^−6∗^	-3.637	0.0009^∗^	0.832	0.4093
CA3-head	5.833	0.0048^∗^	-3.315	0.0021^∗^	-2.893	0.0067^∗^	0.32	0.7501
CA3-body	10.453	0.0001^∗^	-5.043	1.2 × 10^−5∗^	-3.636	0.0009^∗^	-0.526	0.6011
CA4-head	11.633	0.0001^∗^	-4.639	4.3 × 10^−5∗^	-4.02	0.0003^∗^	0.11	0.9128
CA4-body	14.412	7.5 × 10^−6∗^	-5.496	3.01 × 10^−6∗^	-4.267	0.0002^∗^	-0.726	0.4712
HIPPO-fissure	5.882	0.0046^∗^	-3.543	0.0011^∗^	-2.753	0.0095^∗^	-0.258	0.7974
PRE-head	6.999	0.0018^∗^	-3.727	0.0006^∗^	-3.238	0.0027^∗^	-0.195	0.8461
PRE-body	1.731	0.1856	-1.816	0.0776	-1.6	0.1192	0.114	0.9094
PARA	0.352	0.7048	0.985	0.3309	0.522	0.6051	0.348	0.729
ML-head	17.323	1.1 × 10^−6∗^	-5.249	6.5 × 10^−6∗^	-5.408	5.5 × 10^−6∗^	0.991	0.3265
ML-body	16.79	1.6 × 10^−6∗^	-5.466	3.3 × 10^−6∗^	-5.126	1.3 × 10^−5∗^	0.562	0.5767
GC-ML-DG-head	12.932	2.1 × 10^−5∗^	-4.753	3.02 × 10^−5∗^	-4.467	8.8 × 10^−5∗^	0.552	0.5831
GC-ML-DG-body	13.523	1.4 × 10^−5∗^	-5.299	5.6 × 10^−6∗^	-4.273	0.0002^∗^	-0.161	0.8724
FIM	8.264	0.0007^∗^	-3.389	0.0017^∗^	-4.198	0.0002^∗^	0.408	0.6852
HATA	2.046	0.1381	-1.451	0.1551	-2.069	0.0465^∗^	0.772	0.4435
Whole_HIPPO_head	15.012	4.99 × 10^−6∗^	-4.959	1.6 × 10^−5∗^	-4.948	2.1 × 10^−5∗^	0.811	0.4208
Whole_HIPPO_body	17.137	1.2 × 10^−6∗^	-5.467	3.3 × 10^−6∗^	-5.228	9.4 × 10^−6∗^	0.19	0.8503
TAIL	12.332	3.2 × 10^−5∗^	-4.763	2.9 × 10^−5∗^	-4.619	5.6 × 10^−5∗^	-0.229	0.8198
Whole_hippocampus	17.735	8.2 × 10^−7∗^	-5.597	2.2 × 10^−6∗^	-5.392	5.8 × 10^−6∗^	0.44	0.6617

**Table 6:** ANOVA test and multiple comparison results of the subregion volumes of the left amygdala between the three groups.

Left	AD-MCI-NC		AD vs. MCI		AD vs. NC		NC vs. MCI	
	*F* value	*p* value	*T* value	*p* value	*T* value	*p* value	*T* value	*p* value
La	2.743	0.072	-1.917	0.063	-3.007	0.005^∗^	-0.264	0.793
Ba	3.016	0.056	-2.001	0.053	-3.526	0.001^∗^	-0.42	0.676
AB	5.62	0.006^∗^	-2.691	0.011^∗^	-4.959	2.1 × 10^−5∗^	-0.298	0.767
AAA	4.383	0.017^∗^	-2.47	0.018^∗^	-3.961	3.8 × 10^−4∗^	-0.787	0.435
Ce	10.803	9.6 × 10^−5∗^	-4.094	2.2 × 10^−4∗^	-5.148	1.2 × 10^−5∗^	-0.588	0.559
Me	7.868	0.001^∗^	-3.606	0.001^∗^	-4.084	2.7 × 10^−4∗^	-0.802	0.426
Co	8.951	3.9 × 10^−4∗^	-3.588	0.001^∗^	-4.759	3.7 × 10^−5∗^	-0.19	0.85
CAT	2.1	0.131	-1.574	0.124	-3.67	8.5 × 10^−4∗^	0.025	0.98
PL	1.42	0.25	-1.366	0.18	-2.281	0.029^∗^	-0.122	0.903
Whole	3.597	0.033^∗^	-2.162	0.037^∗^	-3.872	4.8 × 10^−4∗^	-0.315	0.754

**Table 7:** ANOVA test and multicomparison results of the subregion volumes of the right amygdala between the three groups.

Right	AD-MCI-NC		AD vs. MCI		AD vs. NC		NC vs. MCI	
	*F* value	*p* value	*T* value	*p* value	*T* value	*p* value	*T* value	*p* value
La	6.937	0.002^∗^	-3.59	0.001^∗^	-3.067	0.004^∗^	-0.452	0.653
Ba	10.201	1.5 × 10^−4∗^	-4.255	0.0001^∗^	-3.809	0.001^∗^	0.109	0.913
AB	12.907	2.1 × 10^−5∗^	-4.882	2.3 × 10^−5∗^	-4.356	1.2 × 10^−4∗^	0.053	0.958
AAA	13.87	1.1 × 10^−5∗^	-5.005	1.4 × 10^−5∗^	-4.634	5.4 × 10^−5∗^	-0.619	0.538
Ce	10.962	8.6 × 10^−5∗^	-4.192	0.0001^∗^	-4.907	2.4 × 10^−5∗^	0.246	0.807
Me	7.368	0.001^∗^	-3.552	0.001^∗^	-4.033	3.1 × 10^−4∗^	0.061	0.952
Co	15.089	4.7 × 10^−6∗^	-5.624	2.3 × 10^−6∗^	-4.518	7.6 × 10^−5∗^	-0.574	0.568
CAT	6.74	0.002^∗^	-3.393	0.002^∗^	-3.301	0.002^∗^	0.178	0.859
PL	4.339	0.017^∗^	-2.861	0.007^∗^	-2.47	0.019^∗^	0.198	0.844
Whole	10.724	0.0001^∗^	-4.381	9.3 × 10^−5∗^	-3.913	4.3 × 10^−4∗^	-0.148	0.883

HIPPO: hippocampus; SUB: subiculum; PRE: presubiculum; PARA: parasubiculum; ML: molecular layer; HATA: hippocampus amygdaloid transition area; FIM: fimbria; GC-DG: granule cell layer of dentate gyrus; La: lateral; Ba: basal; AB: accessory basal; Ce: central; Me: medial; Co: cortical; CAT: corticoamygdaloid transition area; AAA: anterior amygdala area; PL: paralaminar nucleus; Ot: optic tract.

## Data Availability

The data set supporting the results of this article are included within the article. The data sets used and/or analyzed during the current study are available from the corresponding author on reasonable request.
